# Seizing opportunities: Object neophobia as a factor mediating technical innovation in Goffin´s cockatoos?

**DOI:** 10.1371/journal.pone.0335028

**Published:** 2025-11-11

**Authors:** Theresa Rössler, Mark O’Hara, Berenika Mioduszewska, Remco Folkertsma, Roger Mundry, Alice M. I. Auersperg

**Affiliations:** 1 Comparative Cognition, Messerli Research Institute, Interdisciplinary Life Sciences, University of Veterinary Medicine Vienna, University of Vienna, Medical University of Vienna, Vienna, Austria; 2 Department of Cognitive Biology, University of Vienna, Vienna, Austria; 3 Cognitive Ethology Laboratory, German Primate Center, Leibniz Institute for Primate Research, Göttingen, Germany; 4 Leibniz Science Campus Primate Cognition, Göttingen, Germany,; 5 Georg-August-Universität Göttingen, Johann-Friedrich-Blumenbach Institute, Department for Primate Cognition, Göttingen, Germany; University of Tuscia, ITALY

## Abstract

Most studies find substantial inter-individual differences in problem-solving performance within a species, which can drastically influence an individual´s fitness. It has been suggested that innovative behavior can be strongly affected by behavioral traits, such as exploration, persistence, neophilia, and neophobia. Individuals who are more neophobic than others may encounter fewer opportunities or favorable interactions but may also show differences in cognitive ability. We tested eleven captive Goffin’s cockatoos (*Cacatua goffiniana*), a model species for innovative problem-solving, for individual variation in object neophobia and whether it relates to motivation and performance in a problem-solving task. We found no evidence that the less neophobic cockatoos were generally better problem-solvers, nor that they were more eager to engage with the setup after familiarization. Our results suggest that object neophobia in this group of Goffin’s cockatoos may not be directly linked to either the ability to innovate or the general motivation to interact with experimental apparatuses. We discuss what these findings could imply for the emergence of innovative behavior as well as the potential limitations of individually tested animals of a highly social species.

## Introduction

Why are some individuals better innovators than others? Many studies on innovative problem-solving have found substantial differences in performance not only between but also within a species (for an overview, see meta-analysis in [1]). Whereas innovations can differ in kind (e.g., technical or social innovations) and can occur across different contexts (e.g., during predator defense or foraging), it is unclear whether different types of innovations are based on similar mechanisms. Foraging innovations in wild populations are so far most thoroughly studied in birds (e.g., [2–10]) and primates (e.g., [11–13]). Early notable examples include the emergence of novel foraging behaviors on  anthropogenic food sources, such as the milk-bottle opening in great tits (*Parus major*) [14] or the potato washing in Japanese macaques (*Macaca fuscata*) [15]. Other cases involve the sudden appearance of tool use in species that are not specialized, species-wide tool users, such as long-tailed macaques (*Macaca fascicularis*) utilizing stones for masturbation [16] or Bruce a kea parrot (*Nestor notabilis*) using pebbles for feather care [17] (for a review, see [18]). In experimental settings, problem-solving performance on puzzle boxes or obstacle removal is often used as a means to estimate the innovative abilities of an animal (reviewed in [19]).

Innovative problem-solving, i.e., “finding solutions to novel problems or new solutions to familiar problems” [12], can facilitate overcoming obstacles or accessing new resources and can result in increased fitness (e.g., [9,20–22]; but see [23]). The suggested mechanisms underlying innovative behavior and problem-solving ability are manifold. They include behavioral traits such as tendencies for exploration, persistence, motivation, and approach/avoidance patterns in the presence of novel objects (see [19,21]). These traits can mediate (facilitate or inhibit) innovative behavior at different phases of the process ([22]; discussed below). Moreover, they may correlate with other behaviors to form a ´behavioral syndrome´ (see [24]), or may affect an individual´s cognition [25–28], for example during learning [29–32] or problem-solving (e.g., [20,33–36]).

Beyond individual behavioral traits, a species’ ecology is also believed to influence its tendency to innovate. For instance, opportunistic feeding generalist, who have to cope with sometimes unpredictable environmental changes, tend to express more innovative behavior than highly specialized species [37,38]. In particular technical innovations and tool use are more commonly observed in animals, who exhibit extractive foraging [39,40] and are inclined to manipulate and (re-)combine objects [41–44]. Moreover, technical/kinematic foraging innovations are found to positively correlate with brain size [9,45] and the number of pallial neurons [46]. Still, it is important to note, that not every innovation event is necessarily linked to sophisticated forms of cognition; species-predispositions, such as morphology or motor diversity, can have a drastic impact on the resulting innovative behavior (see, e.g., [37,47,48]). Our understanding of the influence of social context on the expression of innovative behavior is still limited (but see, e.g., [49,50]). However, social context can potentially be influential; for example by overcoming initial neophobia [51] or through collective problem-solving [52].

Goffin’s cockatoos (*Cacatua goffiniana*; short: ‘Goffins’) are highly social, opportunistic feeding generalists [53,54] and are skillful problem-solvers when confronted with technical tasks. However, they also show substantial individual variation in their performance (e.g., [43,55]). Probably most notable are innovations of tool use and manufacture, only exhibited by some individuals (hand-raised: [56]; wild: [57]). Captive-raised Goffins can socially learn to use tools [58], yet they use drastically different techniques to handle the tools [58,59]. In the current study, we investigated whether and at what level among-individual variations in certain behavioral traits mediate the problem-solving performance of individuals in a captive group of Goffin’s cockatoos.

Inherent in the definition of innovation itself is the concept of novelty (e.g., “a new idea, method, or device” [60]). To understand the innovative process, it is therefore crucial to investigate how an agent perceives and reacts to encounters with novelty. Like innovation, responses to novelty can vary across different contexts (for a review, see [61]). There are multiple established testing paradigms to estimate neotic style (i.e., approach and avoidance tendencies to novelty), however, they measure different types of novelty responses and are therefore not fully interchangeable. It is thus important to choose a test that is suitable for addressing the research question at hand (as discussed in [61]). Since technical/kinematic innovations require an agent to physically engage with objects, responses to novel objects may be the most relevant type of novelty response to affect technical problem-solving.

Regarding the operationalization of these phenomena, the initial responses to novelty are most commonly categorized as attraction/approach (neophilia) or aversion/retreat (neophobia; e.g., [62–66]). Although these categories intuitively seem to be opposites, they are not necessarily the ends of a single scale, but rather separate phenomena driven by different selection pressures [67,68] and may be regulated by different genes [69,70]. Neophobia and neophilia can thus function independently (as described in the 2-factor model after [71]). A commonly used testing scheme for object neophobia has been established by Mettke-Hofmann and colleagues [67]: the latency to feed next to a novel object is compared to the latency to feed without the object (for a review of variations of the test paradigm, see [61]; for earlier studies involving other novel object tests, see [72,73]). Neophilia on the other hand, is often measured in latencies to approach novelty without a food incentive and/or the duration of haptic exploration of a novel object (e.g., [38,67,74,75]). Thus, explorative behavior is often operationalized to measure neophilia. For the purpose of this study, we grouped exploratory behavior with general motivation to engage with objects/tasks (such as the number of physical contacts) that persists after habituation, i.e., after repeated visual exposure to the apparatus.

A framework to study innovation was proposed by Tebbich and colleagues [22]. It suggests phases of the innovative process and for each, addresses the mechanisms and possible functions. According to this framework, the most critical phases in which novelty can affect the innovative process are: (1) the discovery of an opportunity (e.g., yet unexploited resources), and (2) finding a favorable interaction with it (e.g., a new technique to exploit this resource). Alternatively, neotic responses might be more generally linked to the cognitive ability to innovate (e.g., quickly learning to repeat an accidental beneficial discovery). On a taxonomic level, species that show high sensitivity to novelty (both in approach and avoidance) often show high innovation rates and problem-solving abilities (reviewed in [51]). However, studies examining the relationship between among-individual variation in reactions to novelty and problem-solving ability have resulted in mixed findings [8,20,33,34,36,76–78]. To investigate individual neophobia in Goffin’s cockatoos, we conducted a novel object test and examined whether among-individual variation correlated with either their motivation to engage or their performance in a previously conducted problem-solving experiment, the Innovation Arena [79,80].

The Innovation Arena was developed to further within- and between-species comparisons of innovative problem-solving and to provide the means to compare innovation rate per time unit within the setup. The Arena consisted of 20 simultaneously presented baited problem-solving tasks, to which the subjects were repeatedly exposed. Before the birds were confronted with the Arena, they were habituated to the non-functional apparatus, thus keeping potential neophobic reactions to a minimum. Individual Goffin’s cockatoos varied considerably in their performance [79,80]. Their problem-solving success was influenced by a grouped variable (the frequency of contact with baited tasks as well as with solved tasks, time spent in proximity of the tasks, and how many of the 20 tasks were touched). Since all were related to the motivation to engage with the task, ‘motivation’ was used as an umbrella term for this variable. This now presents an opportunity to investigate to what extent individual neotic style is part of this motivational syndrome and/or whether it has a direct relationship with the cognitive ability to solve technical foraging problems.

In the current study, we used nine different objects to evaluate whether individual neophobic responses are consistent. Each bird was first confronted only with food to control for general motivation and subsequently confronted with food next to a novel object in 10 consecutive trials. In case a subject did not consume the reward within one minute of the control trial, we aborted the session for this testing day. By doing so, we verified that the individual was generally motivated to approach and eat the food. Moreover, by implementing multiple trials with the same novel objects, we were able to test whether observed differences in latency to approach were due to the novelty of the object. If a difference in latency to approach the food in test trials versus control trials was affected by an individual´s neophobia towards the object, we expected approaches to become quicker with repeated exposure.

We evaluated possible influences on object neophobia, the variation and consistency regarding individuals and objects, and whether latencies to approach declined after repeated exposure. Moreover, if neotic style is part of a broader motivational trait, one would expect a correlation between neophobia and measures of motivation in the context of the problem-solving task. Lastly, we investigated whether the neotic style affected general differences in problem-solving ability. If so, less neophobic individuals should outperform more neophobic individuals in the problem-solving task even after they were thoroughly habituated to the setup. If not, we would conclude that the influences of neophobia in this group of Goffin’s cockatoos are limited to the likelihood of approaching and/or interacting with opportunities rather than problem-solving ability.

## Materials and methods

### Subjects and housing

We tested 11 birds (4 females, 7 males; 7–10 years of age) within a six-month period (for details about the exclusion of two additional individuals, see ‘S1 Supplementary Material’). All birds were housed in a large group aviary (heated indoor area: 45m^2^, height: 3-6m; outdoor area: 150m^2^, height: 3-6m) equipped with enrichment (including new objects that were introduced at irregular intervals). The subjects were individually identifiable by colored rings, except one subject (Konrad) that was reliably identifiable through visible body differences (missing two toes on his right foot). The cockatoos were fed a variety of fruits, vegetables, seeds and dietary supplements. They were regularly tested in behavioral and cognitive studies, which involved the handling of objects or the manipulation of an apparatus (for detailed information on each individual´s experimental history, see ‘S1 Supplementary Material’). A previous experiment has investigated neotic style of individuals of the same group [81], however the experiment was conducted on a touchscreen and did thus not involve physical novel objects.

### Novel objects

Novel objects were chosen to differ in color, texture, size, geometric form, and complexity. All were artificial objects that were not encountered in the aviary as enrichment and did not resemble living organisms. We deliberately used artificial objects with randomly varied attributes, as our focus was to measure the general reaction to novelty, rather than to identify which specific object features elicit the strongest neophobic reactions in Goffin’s cockatoos (but see [82]). However, each subject was confronted with the same nine objects that were of three different sizes (measured along the longest side: small (S; 5 cm), medium (M; 15 cm), and large (L; 25 cm). We randomly assigned half of the birds to start with objects of the category small (group ‘small’) and continued with medium and then large objects. Subsequently, the same size order was repeated with the next set of objects until all nine were presented. Conversely, the other half of the subjects (group ‘large’) were presented with objects in the following order: L, M, S, L, M, S, L, M, S. Objects were assigned randomly to each bird within their respective size category. Group identity was balanced with regard to sex.

### Novel object test

#### Setup.

**Fig 1:**
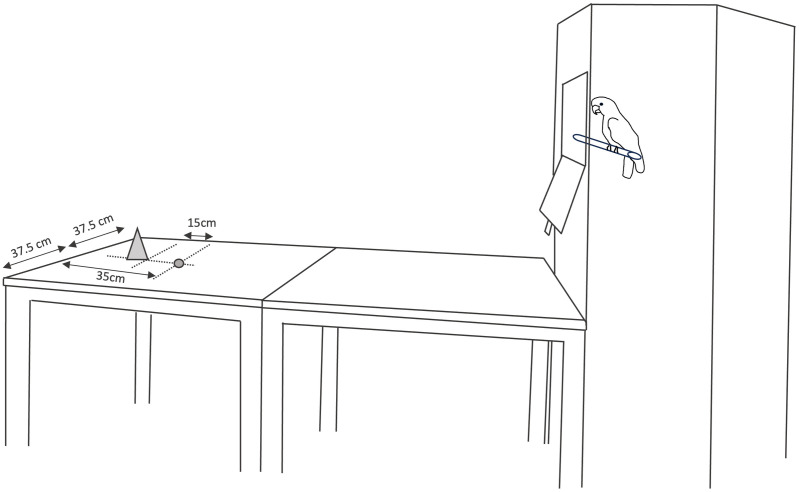
Camera angle and schematic overview of test setup in neophobia trials. The grey circle depicts the reward (35 cm from the end of the table), whereas the grey triangle represents the object (outer rim at a distance of 15 cm from the reward); dotted lines indicate measurement lines; the cockatoo is sitting at the start position inside the cage with the door already opened.

The birds were tested in visual isolation from the group in the adjacent test compartment. Two identical tables (75cm^2^ surface area each) were positioned next to each other resulting in a rectangular table (Fig 1). At the end of one of the shorter sides of the tables was a large cage. Food was always placed 35 cm from the shorter side of the table opposite the cage (see ‘Habituation’ for an exception). In neophobia trials, the objects were positioned such that the outer rim was at a 15 cm distance from the food at the distal end of the tables (from the perspective of the cage). All trials were recorded with a digital camcorder positioned next to the long side of the tables.

#### General procedure.

The experimenter (TR) asked individual birds to enter the test compartment by calling them by name. The subject was then positioned on a perch inside the cage and the door closed. In control trials, the experimenter showed the reward to the bird saying “Nüsschen” (“small piece of nut” in German), placed the food (a small piece of cashew) on the table, and shortly placed the finger next to it. This was done to ensure that the subject saw the food placement. Then the cage door was opened, and the bird was free to approach and consume the food. In neophobia trials, the object was positioned first, followed by the same procedure as in control trials. In all trials, the experimenter would walk away from the cage door and take a seat at the opposite end of the test chamber. After each trial, the subjects were asked to go back into the cage (two birds received a slightly modified setup as they were not habituated to the cage; for details see ‘S1 Supplementary Material’). The experimenter (TR) has been familiar with the birds for approximately two months prior to the onset of testing.

#### Habituation.

The birds were fed small pieces of cashew and sunflower seeds in the cage and on both tables, as well as after taking them in and out of the cage and closing/opening the cage door, until they showed no behavioral indicators of discomfort (e.g., raised crest or spread tail feathers) during the procedure, readily entered and exited the cage and fed on the provided treats. To ensure that any potential neophobic reactions would be solely elicited by the novel object, we additionally habituated them to the procedure before the first test sessions. Food was placed at the center of the table closest to the bird and trials were repeated until subjects consumed the food within one minute after opening the cage door in three consecutive trials.

#### Test protocol.

Subjects received one test session with one novel object per test day. Each session consisted of one control trial (food only) followed by 10 neophobia trials (novel object and food). In case the subject did not consume the food reward within one minute of the control trial or within 15 minutes of a neophobia trial, the session was aborted and continued the next test day. Such continued sessions were again initiated by a control trial and then resumed with the neophobia trial in which the dropout occurred (almost exclusively in neophobia trial 1). For example, if a session had to be aborted because the time limit was exceeded in neophobia trial 3, the continued session would start with one control trial followed by neophobia trials 3–10. To avoid an artificial cut-off time and a potential overestimation/inflation of repeatability as a result, we only proceeded to the next object if the food was consumed in all trials. After the last neophobia trial, the experimenter gave the birds five uninterrupted, additional minutes with the object (‘exploration phase’). As the birds rarely showed interest after consuming the reward, we did not include these data in the analysis. Minor deviations from the procedure and protocols for necessary interventions by the experimenter can be found in ‘S1 Supplementary Material’.

### Problem-solving task (Innovation Arena)

We used a subset (which only included birds that participated in both experiments) of a dataset from a previously published problem-solving experiment [79] that was conducted after the data collection of the novel object test. In said experiment, the same subjects were individually tested in the Innovation Arena, which consisted of 20 different tasks arranged in a semi-circle. Each individual was given 20 minutes per session to solve as many tasks as possible. Sessions were repeated (one per test day) until no new tasks were solved in 5 consecutive sessions or no task was solved for 10 successive sessions. Importantly, testing was preceded by a thorough habituation to the Arena to eliminate neophobia towards the setup (for detailed protocols, see [79,80]). During habituation, individual birds were first required to insert their head into each box of the ‘empty’ Arena (i.e., all acrylic glass boxes attached to a wooden platform but without functional parts and front panels) to retrieve a food reward (criterion: consume all 20 rewards within 10 minutes in 3 consecutive sessions). Birds were additionally habituated to single, non-attached task elements that possibly could have evoked neophobic reactions. Moreover, they were confronted with the fully functional Arena but baited on top of each box instead of inside (criterion: consume all 20 rewards within 10 minutes in 1 session), to ensure that they were fully habituated to the final visual appearance of the Arena. Only after reaching both criteria, did the testing begin. A minimum of three months separated the final novel object test from the first individual habitation in the test compartment, and at least four months passed between the final novel object test and the first problem-solving test session. Apart from recording the number of found solutions, we also coded several variables of task-directed behaviors: number of contacts with baited tasks, number of contacts with already solved tasks, active time spent within 20 cm of the tasks, latency to approach the tasks within 20 cm, how many of the 20 tasks were touched, and the number of tasks touched but not solved (for more details, see [79,80]).

### Behavioral coding

We extracted latencies to touch the food from the recorded videos using BORIS (Behavioral Observation Research Interactive Software; [83]). Latency measurement began when the experimenter removed their hand from the door and ended when the bird first touched the food. One video (Pipin, object 3) was lost and thus could not be analyzed. A second coder, not otherwise involved in the study, coded approximately 5% of the sessions. Inter-observer reliability was excellent (ICC(C,1)=1; *p* < 0.001; see ‘S2 Supplementary Results’ for more details).

### Statistical analysis

In the case of aborted neophobia trials that were later resumed, we used the cumulative duration for the analysis. When a bird received multiple control trials per object (resumed sessions also started with a control trial), we used the mean value for the analysis. However, control trials in which the individual did not touch the food within the first minute were excluded, as no corresponding neophobia trial was conducted on those days. Control trials functioned as a measure of the individual speed of approach per day. To investigate whether neophobia affected problem-solving or motivation to interact with the problem-solving task, we used the data of the Innovation Arena from the individuals who participated in both experiments.

#### General remarks.

We fitted multiple generalized linear mixed models along with other statistical tests (details below). All checks of relevant model assumptions can be found in ‘S1 Supplementary Material’. Statistical output including estimates, standard errors/deviations, test statistics, confidence intervals and model stabilities (obtained by dropping individual levels of random effects factors, one at a time [84]) are provided for each model in ‘S2 Supplementary Results’. We compared each full model to a respective null model (lacking the term(s) of interest) using likelihood ratio tests [85]. In each model all theoretically identifiable random slopes were included [86,87]. To avoid ‘cryptic multiple testing’ [88] significance tests of individual predictors or interactions were only conducted if the full-null model comparison revealed a significant effect. Given that interactions within a model were not significant, we fitted reduced models without the interaction terms to obtain fixed effects estimates unconditional with respect to other terms in the model. All final model structures and results as well as variable transformations and reference levels can be found in ‘S2 Supplementary Results’.

#### Effects on the latency to feed in neophobia trial 1.

The first model was used to test the influence of multiple predictors as well as the effect of individual and object on the latency to feed next to the novel object. We used latency to feed in the first neophobia trial as the response variable and four fixed effect predictors: the latency to feed in control trials, object size (small, medium, large), group (starting with small or large objects), and object sequence (1–9). We expected that the latency to feed might be differently influenced by object sequence and object size depending on group identity, and therefore we additionally entered two-way interactions between those variables. Furthermore, we included random intercepts for both individual and object. As the response variable was bound at zero, we used a gamma error distribution with log link function. We encountered convergence issues with the specified model and therefore removed the correlations among random intercepts and random slopes.

Testing random effects via likelihood ratio tests is controversial due to boundary issues and undefined degrees of freedom (see, e.g., [89]). Therefore, we additionally fitted a Bayesian model with the same structure and gamma distribution to validate our likelihood ratio test results by investigating 95% credible intervals for the random effects.

#### Individual neophobia score.

We used the above-described model 1 to estimate individual neophobia scores based on the random intercept of each individual. This approach allowed us to extract variance components directly form the model, thereby accounting for other influential factors, such as the effect of object identity. In other words, the model incorporates the influence of control trial latencies, similar to the commonly used ratio between control and neophobia trial latencies but is less affected by additional confounding variables. We believe this provides a more robust measure of neophobia. Moreover, it enables us to apply the ‘double-adjusted’ repeatability measures described in: ‘Repeatability of individuals and objects’.

Thus, we extracted the estimates of the individual-specific random intercepts as neophobia scores. As the model also estimated random slopes within individual (latency in control trial, object size, and object sequence), the random intercept value depended on the group mean of each predictor that entered the model as a random slope (due to centering and z-transformations). However, estimates might change differently for individuals along the random slope gradients, possibly resulting in varying differences between individuals (as discussed in [90]). To address this issue, we evaluated each random slope for consistency of individual differences over the entire range of the respective predictor. Individual-specific slopes of latency to feed in control trials and object size were estimated to change in the same magnitude and direction for all individuals, whereas we found individual differences in the effects of object size. We therefore determined individual-specific fitted values with respect to the random intercept of individual and the mean value for random slope of object size within individual (based on Best Linear Unbiased Predictors; [90,91]). The final scores as well as raw data for comparison can be found in ‘S2 Supplementary Results’.

#### Repeatability of individuals and objects.

To estimate repeatability of individuals and objects, we followed the recommendations by [92] for models with a gamma distribution, using the trigamma function to calculate residual variance. Furthermore, we used adjusted/controlled intra-class-correlations (ICC). In such calculations, certain variance components that are not of interest are controlled, i.e., adjusted for, by excluding them from the denominator (see, e.g., [92]). Typically, adjusted ICCs control for the variance of fixed effects resulting in values that concern only the random effect´s and residual variance. As our model included two random intercepts, we further excluded the variance of one to calculate the controlled (or ‘double-adjusted’) intra-class correlation for the other random effect separately. We estimated 95% confidence intervals using parametric bootstrapping (number of bootstraps = 1000). Formulae of all ICC and R^2^ calculations can be found in ‘S1 Supplementary Material’.

#### Trials until habituation.

In the next step, we investigated how quickly the birds habituated to the novel objects with repeated exposure. We first fitted a similar model as model 1, which investigated the effects on the latency to feed in neophobia trial 1, but now predicting latency to feed in all trials. Neophobia trial (1–10) was added as predictor variable. Given a significant effect of trial number in a full-null model comparison, we further ran post-hoc comparisons using signed-rank Wilcoxon tests to determine when the latency to feed significantly decreased. With this, we compared the latencies in each neophobia trial with the latency to feed in the control trials and used Holm’s-correction [93] to adjust *p*-values for multiple comparisons.

#### Principal component analysis of the problem-solving data.

Before including data from the problem-solving study in the subsequent models, we first checked for correlations between the task-directed behaviors, as in the original analysis of the Innovation Arena [79]. The variables considered were: the number of contacts with baited tasks, the number of contacts with solved tasks, the number of tasks touched (out of 20), and the logarithm of the latency to approach the tasks within 20 cm. As they were highly correlated (Barlett’s test: 531.572, k = 6, p < 0.001), we used a Principal Component Analysis (PCA) with orthogonal rotation (only including the eleven birds that participated in both studies). The analysis revealed one principal component with an Eigenvalue above 1 as of Kaiser´s criterion [94] explaining 70.3% of the total variance. It was loaded positively with the number of contacts with solved and baited tasks as well as the number of tasks touched (out of 20). Additionally, latency to approach loaded negatively on the component (see ‘S2 Supplementary Results’). As the variables were measuring readiness/willingness to engage with the tasks, we used ‘motivation’ as the umbrella term to refer to the component (see also [79]).

#### Neophobia score as a predictor of motivation.

To answer the question of whether object neophobia in the novel object test and motivation in the Innovation Arena experiment were associated, we fitted a GLMM with a Gaussian error distribution and identity link. The principal component termed ‘motivation’ was entered as the response variable and predicted by neophobia score and session. As the influence of neophobic reactions might vary with the number of sessions (i.e., neophobia may play a larger influence in the beginning), we also included the interaction between neophobia score and session. To avoid pseudo-replication [95], we entered individual as a random intercept [96].

#### Neophobia as a predictor for problem-solving success.

To address the main question of our study, whether object neophobia as an individual trait predicted success in a problem-solving task, we used a binomial model to predict problem-solving performance, measured as proportion of tasks solved. The neophobia score and its interaction with session were entered as predictors, and we controlled for motivation. Individual was entered as random intercept. Lastly, we used a random intercept that combined session per individual to account for day-to-day variation in individual performance.

#### Implementation.

The analysis was conducted using R ([97]; versions 4.3.1. & 4.4.3) in RStudio ([98]; versions 2021.09.0 & 2024.12.1). Several functions used were written by RM, including those for checking model assumptions, model stability and random slope inclusion, as well as a wrapper for bootstrapping utilizing the function ‘bootMer’ of the package ‘lme4’ ([99]; version 1.1−33). We fitted gamma models with the package ‘glmmTMB’ ([100]; version 1.1.7) and the binomial model with ‘lme4’ ([99]; version 1.1−33). *P*-values were determined using likelihood ratio tests [101] with the drop1 function (of base R). The Gaussian model was fitted with ‘lme4’ ([99]; version 1.1−33) and ‘lmerTest’ to extract *p*-values ([102]; version 3.1−3; as recommended by [103]). The package ‘car’ ([104]; version 3.1−2) was used to determine Variance Inflation Factors (VIF; [105,106]) to check collinearity among *p*redictors. The Bayesian model was fitted with the package ‘brms’ ([107]; version 2.22.0). To avoid package conflicts during model compilation, we executed this model in a clean R environment using the ‘callr’ package ([108]; version 3.7.6). We used the ‘posterior’ package ([109]; version 1.6.1) to extract posterior samples and credible intervals. Plots were generated using ‘ggplot2’ ([110]; version 3.5.2), ‘ggbeeswarm’ ([111]; version 0.7.2), ‘ggpubr’ ([112]; version 0.6.0), ‘gridExtra’ ([113]; version 2.3), ‘cowplot’ ([114]; version 1.1.3), and ‘ggimage’ ([115]; version 0.3.3). We checked the correctness of manual extractions of variances using the ‘insight’ package ( [116]; version 0.19.3) and used the package ‘rela’ ([117]; version 4.1) for PCA. The packages ‘irr’ ([118]; version 0.84.1) was used to test inter-observer reliability. Additionally, we used the package collection ‘tidyverse’ ([119]; version 2.0.0) and ‘dplyr’ ([120]; versions 1.1.2 & 1.1.4) for data management and the packages ‘conflicted’ ([121]; version 1.2.0) to handle function name conflicts.

#### Ethics statement.

All cockatoos involved in this study were bred by certified European breeders and have CITES documentation. In accordance with the Austrian Animal Protection Act (§ 25 – TschG. BGBl. I Nr. 118/2004 Art. 2. 118) they are registered at the district’s administrative animal welfare bureau (Bezirkshauptmannschaft St. Pölten Schmiedgasse 4–6, 3100; St. Pölten, Austria). Participation in the study was voluntary (subjects were asked to enter the test compartment by calling them by their name) and non-invasive. Therefore, the presented study was not classified as an animal experiment by Austrian Law (TVG, 2012). The Ethics and Animal Welfare Committee of the University of Veterinary Medicine Vienna approved the study (ETK-027/02/2022).

## Results

**Fig 2:**
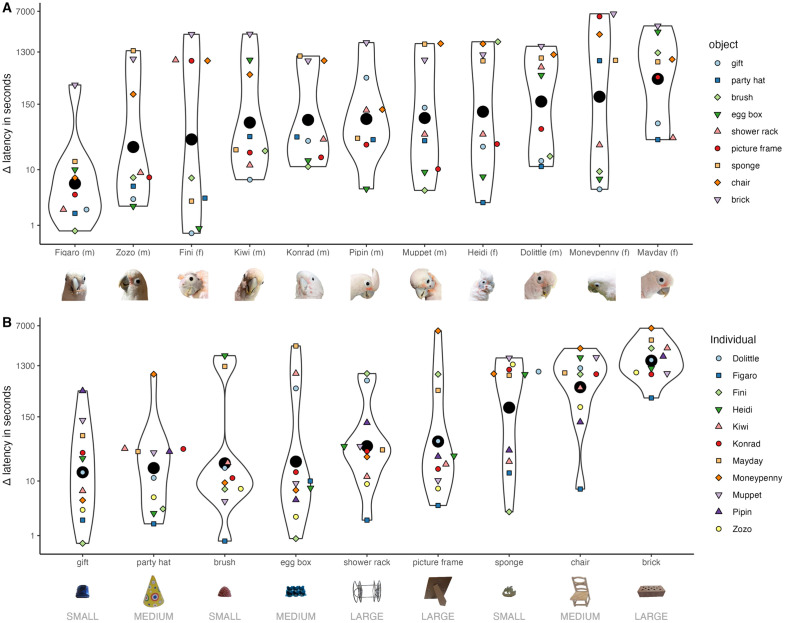
Difference (Δ) in latency to feed per individuals and objects (log-transformed data). Colors and shapes depict values A) per object, B) per individual; the black circles indicate group mean; the violin shapes show group distribution; x-axes are ordered by group average. The size category of each object can be found below object names. Sex of the individual birds is denoted by ‘f’ (female) or ‘m’ (male) in parentheses. Images are not to scale.

The subjects were generally motivated to feed within the first minute of the control trial and only failed to do so in three sessions (three birds, once each). Individual differences had significant effects on the latency to feed (full model compared to reduced model lacking the random effect ‘individual’: 𝜒^2^_(5)_=44.684, p < 0.001; SD = 1.391; Fig 2). This finding was corroborated by an informal significance test based on the Bayesian model, as not a single posterior sample of the random intercept of individual was zero and the lower 95% credible limit was estimated to be 0.859. The double-adjusted intra-class correlation as a measure of repeatability within individuals was estimated to be r = 0.654 (95% CI = 0.351–0.743 for all converged models; all ICC and R^2^ results can be found in ‘S2 Supplementary Results’).

Furthermore, the identity of each object similarly affected latencies to feed (full model compared to reduced model lacking the random effect ‘object’: 𝜒^2^_(5)_=62.094, *p* < 0.001; SD = 1.249 Fig 2; Bayesian model: no posterior sample zero; lower 95% credible limit estimated at 0.976), and this effect was similarly consistent to that observed for individual (double-adjusted r = 0.604 95% CI = 0.294–0.712 for all converged models).

**Fig 3:**
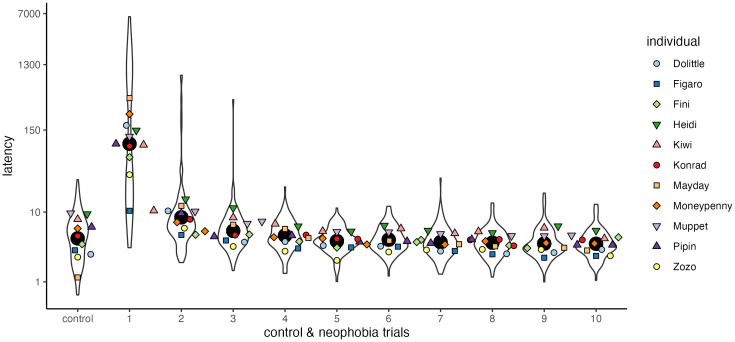
Latencies to feed in control trials and repeated exposure to the same objects (neophobia trials 1-10; log-transformed data). Colors and shapes depict mean values per individual; black circles indicate group mean; violin shapes show group distribution.

The full-null model comparison with respect to the predictors object size, group identity and object sequence and their interactions did not reveal a significant effect (𝜒^2^_(7)_=9.446, *p* = 0.223). When the birds were confronted with the same object next to the food, they quickly habituated after the first times they fed (−1.053 + /-0.164, 𝜒^2^_(1)_=17.557, *p* < 0.001; Fig 3). Notably, post-hoc tests showed that the latencies in neophobia trials were already not significantly different from those in control trials in neophobia trial 3 (mean = 10 sec, SD = 40.80, *p* = 0.356).

**Fig 4:**
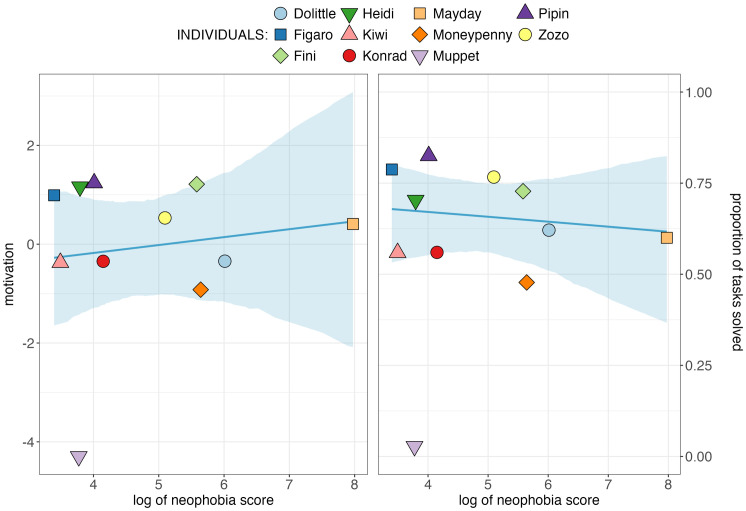
Object neophobia in relation to (a) the principal component ‘motivation’ (left) and (b) problem-solving success (right). Colored shapes depict values per subject. Blue line shows fitted values of model and blue shade represents 95% confidence intervals.

We found no evidence that the neophobia score extracted from the novel object test predicted motivation in the problem-solving task when removing the fixed factor and its interaction from the model (𝜒^2^_(2)_=3.408, *p* = 0.182; reduced model estimate + /- SE = 0.302 + /-0.438; see Fig 4). Additionally, individual object neophobia failed to predict problem-solving success (𝜒^2^_(2)_=0.174, *p* = 0.917; reduced model estimate + /- SE = −0.074 + /-0.196; see Fig 4). For more details on statistical output, confidence intervals and model stabilities see ‘S2 Supplementary Results’).

## Discussion

Innovative problem-solving is influenced by multiple factors at different stages of the innovative process [19,21,22,36,122]. We investigated whether and to what extent individual problem-solving success in Goffin’s cockatoos is affected by variation in neophobia, either at the stage of finding new opportunities, when finding a favorable interaction (i.e., motivation to engage with the task), or whether it is directly linked to a subject´s ability to innovate.

We found no evidence that the less neophobic individuals in our group of Goffin´s cockatoos were better problem-solvers, nor that neophobic tendency was linked to task-directed behaviors in general. This suggests that neophobia may primarily influence the initial phase of identifying opportunities to innovate. We will first elaborate on the results of the novel object test, then discuss the implications of our findings on how neophobia may influence the innovative process.

The variation of neophobic responses, i.e., the latency to feed next to novel objects, was consistent within individuals (double-adjusted r = 0.654) and higher than in previous studies (meta-analyses for responses to novel objects: r = 0.47 [123] and behaviors in general r = 0.37 [124]). However, this comparison should not be overinterpreted as different calculation methods were used in different studies.

Our setup accounted for food motivation: we first ensured that birds were generally interested in eating the reward quickly (by setting a criterion in control trials). We confirmed that increased latencies in neophobia trials were strongly influenced by the novelty of the object: the latencies to feed next to the same object decreased after the first consumption of food and repeated exposure, i.e., with decreasing novelty, hence familiarization with the object. Repeated trials and therefore repeated consumption of rewards could theoretically have led to a satiation effect as sessions progressed. However, the drastic decrease and the consistently short latencies observed up to the final neophobia trial (see Fig 3) suggest that any potential effect is unlikely to be substantial. Note that for the calculation of the neophobia score we only used the first encounter with the novel object in our analysis.

Controlling for the level of novelty that each object represents for each individual is more challenging. We purposefully chose a variety of different shapes and colors to avoid conclusions based on specific object properties (with the exception of size). The reaction towards specific objects was consistent on a group level, meaning that single objects elicited similar latency trends in different individuals (double-adjusted r = 0.604), and had a similar influence as the identity of the subjects on the overall latency to feed. Most of the birds were acquired shortly upon weaning and kept in the same aviary and hence were exposed to the same enrichment. Four birds were already three to four years old when they joined the group, but they had previously been kept in a breeder’s aviary (rather than as pets in a human household), where they had access to wooden parrot toys that are commonly used as enrichment in parrot husbandry. Therefore, it can be assumed that the subjects who participated in this study had fairly similar experiences both with enrichment objects in the aviary and items used in experimental tests (for more information on each individual´s experimental history, see ‘S1 Supplementary Material’). Thus, we assume that the variation between individuals is unlikely to be attributed to the objects representing different levels of novelty to each subject.

We found no evidence for a strong combined effect of object size, object sequence and group (i.e., whether the birds were first presented with the large or the small object). This is particularly surprising in relation to object size, as it contrasts with the findings of a similar study conducted later on the same group of Goffin’s cockatoos, involving largely the same individuals (10 out of 11) [82]. To discuss potential reasons for this difference in findings we first elaborate on the similarities and differences of the two studies: The present study aimed to test for individual variation in object neophobia and the connections to overall motivation and problem-solving ability rather than for the specific aspects of an object that may elicit neophobic reactions. The variation of different attributes of objects was therefore deliberately chosen to cover a range of potential influences (as discussed, e.g., in [61]). The study by Cespedes-Gonzalez and colleagues [82], however, was specifically designed to systematically test which object properties influence neophobic reactions, focusing on the object´s size, color, reflection, and shape. To test size, they used three differently sized cones. Similar to the study presented here, the objects were categorized as small (5 cm), medium (15 cm) and large (25 cm). However, the lengths corresponded to different aspects of the objects. Whereas in the current study the lengths related to the longest side of objects of different shapes, colors, patterns, and materials, in the other study they were related to the base diameter of otherwise identical cones. Thus, the objects used in the two studies were markedly different in dimensions (e.g., the largest cone had a height of 40 cm). Additionally, the volume in the presented study does not necessarily increase with our categorization of size (see, e.g., the object ‘shower rack’ in Fig 2 that was categorized as ‘large’ due to the largest side but at the same time had very little volume). We thus believe that the two studies are not directly comparable. Cespedes-Gonzalez and colleagues [82] found a significant effect when they compared the latency to feed next to the smallest and largest object (but not when either was compared to the medium size). We conclude, that whereas in the current study we did not find an effect of size (as maximum length of an object), it is still likely that Goffin’s cockatoos react more neophobic when confronted with substantially larger objects than smaller ones. This effect might only be apparent given a substantial difference in size and volume. Furthermore, we acknowledge that the model used to investigate this effect was relatively complex in relation to the sample size. As this was inherent to the experimental setup, we suggest that future studies interested in these factors consider a simplified design to reduce the number of predictors.

Regarding our main research question, we found no evidence that consistently less neophobic Goffin’s cockatoos in our group were generally better problem-solvers. This is in contrast to some earlier studies that have investigated this question in different species (for a review, see [26]) but may be explained by crucially different experimental setups: studies finding this correlation typically extracted the variable from the same experiment as problem-solving success (e.g., [20,33–35,125]). However, if protocols included separate tests and habituation to the problem-solving task, this association was not found (e.g., [76,78]). Furthermore, we found no evidence that neophobic tendencies predicted overall motivation to interact with the problem-solving apparatus, which suggests that object neophobia is not part of a larger motivation trait in Goffins. This trait was assessed based on the number of contacts with the apparatus, the time spent in proximity to the apparatus, and the number of tasks touched in the problem-solving experiment and was found to significantly influence problem-solving success. This suggests that neophobia and the general motivation to engage with experimental tasks following initial exposure may operate independently.

Notably, our findings refer to problem-solving after habituation. However, object neophobia may still mediate innovation by affecting the earliest phase of the process (i.e., may be temporally dependent as discussed, e.g., in [81]). Higher neophobia most likely decreases the likelihood of finding an opportunity and consequently of finding a favorable interaction within an early timeframe [22,36]. Conversely, less neophobic individuals might explore earlier and the possibility of finding a favorable interaction, ‘intentionally’ or accidentally, can thus be accelerated [81]. Therefore, innovations may emerge more often in less neophobic individuals in experiments that have time constraints, in fast-changing environments (e.g., quickly perishable or ephemeral resources), or when competing for resources. It is noteworthy, that the individual Goffins typically regarded as the most skilled tool users (Figaro, Fini, Kiwi and Pipin; see, e.g., [59,126]) tended to exhibit low levels of object neophobia in this study (see Fig 2). Future studies may investigate this observed pattern more closely.

Decreased neophobic reactions also encompass risks, such as consuming poisonous food or interacting with a possibly dangerous object/organism [67]. Especially exploratory behavior, which is often exhibited after initial neophobia in animals such as parrots, corvids, or primates, bears the cost of divided attention and consumes time and energy with unreliable benefits [127]. Young individuals are often found to have lower levels of neophobia compared to adults, likely enabling them to explore their environment more readily and to enhance learning [65]. The costs and benefits of neophobia can also vary within an adult individual´s life along multiple factors (such as resource availability, habitat type, or predator abundance; as discussed, e.g., in [61]). Therefore, a more neophobic individual might have an advantage in certain circumstances but a disadvantage in others, which might explain stable among-individual variation in this trait [128].

Goffin’s cockatoos are neophobic, neophilic, and explorative, i.e., they show strong sensitivity to novelty and motivation to interact with novel items [129]. Moreover, both hand-raised and wild individuals showed high levels of innovative problem-solving behavior and pronounced individual differences (e.g., [55,79,130]). Yet, Goffin’s cockatoos are also highly social [53] and both novelty responses and innovations might therefore be strongly influenced by social context. Forss and colleagues [51] extensively reviewed studies on neophobia and exploration in different social contexts and discussed the “paradox of neophobic explorers”, such as corvids and primates. Notably, many of the most innovative species fall in the category of neophobic explorers. Sociality might be key to overcoming neophobia and enabling problem-solving in natural settings [51]. Some studies have found decreased neophobia levels with an increase in group size [34,131–134], whereas others found longer latencies to feed next to novel objects in social than non-social settings [49,135,136].

In this study, we tested subjects individually in both the object neophobia test and the problem-solving task. However, reactions to novelty might be dependent on social context and group composition in Goffins. Although tested without any conspecifics present, we acknowledge that the experimenter might have been perceived as a social partner, despite deliberately avoiding any interaction or response to the bird’s behavior. Future studies may specifically address this question or implement a test protocol that excludes experimenter presence during test sessions.

Individuals might also differ in their plasticity and direction of social influences. For example, some individuals may be very cautious when experiencing novelty alone but one of the first to approach in a group setting, whereas others might always be rather wary. Additionally, individual neophobia might be influenced by the reaction of the social partner. For example, [137] showed that house sparrows (*Passer domesticus*) tested with a partner that was similarly or more neophobic, were more hesitant to approach novel objects than when tested in non-social settings. To understand the pattern of neophobia and its temporal impact on the emergence of innovations in natural settings, future experiments might target the influence of different social compositions on neophobia as well as problem-solving. Nevertheless, the presented study on individually tested Goffin’s cockatoos cautions against interpreting initial aversion as a lack of interest and not, in the words of Greenberg & Mettke-Hofmann [67], to “mistake courage for brains”.

## Supporting information

S1 Supplementary Material Additional information on methods. (PDF)

S2 Supplementary Results Detailed output tables of statistical analysis. (XLSX)

S3 Movie Example video of a test session. Mayday, object 8, session 1. (MP4)

S4 Dataset Novel object. (CSV)

S5 Dataset Innovation Arena. (CSV)
