# Supplementary material for: Seizing opportunities: Object neophobia as a factor mediating technical innovation in Goffin´s cockatoos?
Source: PLoS One. 2025 Nov 11;20(11):e0335028. doi: 10.1371/journal.pone.0335028 (PMC12604780; doi:10.1371/journal.pone.0335028)
Supplement: S1 Supplementary Material — (PDF) [file pone.0335028.s001.pdf]

Full title:

Seizing opportunities: Object neophobia as a factor mediating technical innovation in Goffin´s cockatoos?

Short title:

Limited effect of object neophobia on innovativeness in Goffin's cockatoos

Theresa Rössler[1,2*], Mark O'Hara[1], Berenika Mioduszewska[1], Remco Folkertsma[1], Roger Mundry [3-5], Alice M.I. Auersperg[1]

[1] Comparative Cognition, Messerli Research Institute, Interdisciplinary Life Sciences, University of Veterinary Medicine Vienna, University of Vienna, Medical University of Vienna, Austria

[2] Department of Cognitive Biology, University of Vienna, Austria

[3] Cognitive Ethology Laboratory, German Primate Center, Leibniz Institute for Primate Research, Göttingen, Germany

[4] Leibniz Science Campus Primate Cognition, Göttingen, Germany

[5] Georg-August-Universität Göttingen, Johann-Friedrich-Blumenbach Institute, Department for Primate Cognition, Göttingen, Germany

* Corresponding author

E-mail: theresa.roessler@vetmeduni.ac.at (TR)

SUPPLEMENTARY MATERIAL

EXPERIMENTAL HISTORIES

Table S1: Experimental histories of individuals. Columns show different kinds of experiments participated (physical interaction/touchscreen/neotic style). References can be found to the right of each count. Full references below.

individual	total experiments participated	physical interaction studies	← references	touchscreen studies	← references	neotic style study on touchscreen	← references
Heidi	12	11	[2-6,8-13]	1	[14]	0	-
Moneypenny	13	11	[2-6,8-13]	2	[14,15]	1	[15]
Mayday	12	11	[2-6,8-13]	1	[14]	0	-
Fini	11	10	[2-6,9-13]	1	[14]	0	-
Figaro	15	13	[1-13]	2	[14,15]	1	[15]
Pipin	14	12	[2-13]	2	[14,15]	1	[15]
Konrad	13	11	[2-6,8-13]	2	[14,15]	1	[15]
Dolittle	14	12	[2-13]	2	[14,15]	1	[15]
Kiwi	14	12	[2-13]	2	[14,15]	1	[15]
Zozo	11	11	[2-6,8-13]	0	-	0	-
Muppet	13	11	[2-6,8-13]	2	[14,15]	1	[15]

References for Table S1:

1. Auersperg AMI, Szabo B, von Bayern AMP, Kacelnik A. Spontaneous innovation in tool manufacture and use in a Goffin's cockatoo. Current Biology. 2012;22: R903–R904. doi:10.1016/j.cub.2012.09.002

2. Auersperg AMI, Laumer IB, Bugnyar T. Goffin cockatoos wait for qualitative and quantitative gains but prefer "better" to "more." Biology Letters. 2013;9: 20121092–20121092. doi:10.1098/rsbl.2012.1092

3. Auersperg AMI, Kacelnik A, von Bayern AMP. Explorative learning and functional inferences on a five-step means-means-end problem in Goffin's Cockatoos *(Cacatua goffini)*. Marshall JAR, editor. PLoS ONE. 2013;8: e68979. doi:10.1371/journal.pone.0068979

4. Auersperg AMI, Szabo B, von Bayern AMP, Bugnyar T. Object permanence in the Goffin cockatoo *(Cacatua goffini)*. Journal of Comparative Psychology. 2014;128: 88–98. doi:10.1037/a0033272

5. Auersperg AMI, von Bayern AMP, Weber S, Szabadvari A, Bugnyar T, Kacelnik A. Social transmission of tool use and tool manufacture in Goffin cockatoos (*Cacatua goffini*). Proceedings of the Royal Society B: Biological Sciences. 2014;281: 20140972–20140972. doi:10.1098/rspb.2014.0972

6. Auersperg AMI, van Horik JO, Bugnyar T, Kacelnik A, Emery NJ, von Bayern AMP. Combinatory actions during object play in psittaciformes (*Diopsittaca nobilis*, *Pionites melanocephala*, *Cacatua goffini*) and corvids (*Corvus corax*, *C. monedula*, *C. moneduloides*). Journal of Comparative Psychology. 2014;129: 62–71. doi:10.1037/a0038314

7. Auersperg AMI, Borasinski S, Laumer IB, Kacelnik A. Goffin's cockatoos make the same tool type from different materials. Biology Letters. 2016;12: 20160689. doi:10.1098/rsbl.2016.0689

8. Auersperg AMI, Köck C, Pledermann A, O'Hara M, Huber L. Safekeeping of tools in Goffin's cockatoos, *Cacatua goffiniana*. Animal behaviour. 2017;128: 125–133. doi:doi.org/10.1016/j.anbehav.2017.04.010

9. Laumer IB, Bugnyar T, Auersperg AMI. Flexible decision-making relative to reward quality and tool functionality in Goffin cockatoos *(Cacatua goffiniana)*. Scientific Reports. 2016;6. doi:10.1038/srep28380

10. Laumer IB, Bugnyar T, Reber SA, Auersperg AMI. Can hook-bending be let off the hook? Bending/unbending of pliant tools by cockatoos. Proc R Soc B. 2017;284: 20171026. doi:10.1098/rspb.2017.1026

11. Szabo B, Bugnyar T, Auersperg AMI. Within-group relationships and lack of social enhancement during object manipulation in captive Goffin's cockatoos (*Cacatua goffiniana*). Learning & Behavior. 2016;45: 7–19. doi:10.3758/s13420-016-0235-0

12. Habl C, Auersperg AMI. The keybox: Shape-frame fitting during tool use in Goffin's cockatoos (*Cacatua goffiniana*). Rutherford S, editor. PLOS ONE. 2017;12: e0186859. doi:10.1371/journal.pone.0186859

13. Beinhauer I, Bugnyar T, Auersperg AMI. Prospective but not retrospective tool selection in the Goffin's cockatoo *(Cacatua goffiniana)*. Behaviour. 2018; 1–27. doi:10.1163/1568539X-00003515

14. O'Hara M, Auersperg AMI, Bugnyar T, Huber L. Inference by exclusion in Goffin Cockatoos (*Cacatua goffini*). Boraud T, editor. PLOS ONE. 2015;10: e0134894. doi:10.1371/journal.pone.0134894

15. O'Hara M, Mioduszewska B, von Bayern A, Auersperg A, Bugnyar T, Wilkinson A, et al. The temporal dependence of exploration on neotic style in birds. Scientific Reports. 2017;7. doi:10.1038/s41598-017-04751-0

TESTING PROCEDURE

Exclusion of two subjects (Olympia and Muki)

Two cockatoos were excluded as they did not finish the study in a prolonged timeframe. Olympia participated in 40 sessions but completed only one object, while Muki received 36 sessions and finished five objects. As neither of the two birds took part in the problem-

solving study, they could not yield meaningful data to the analysis of the relationship between problem-solving and object neophobia.

Deviation from test protocol for two subjects (Fini and Muki)

Two subjects (Fini and Muki, who was excluded from the experiment later on) had strong aversions towards the start cage. Therefore, for testing they were allowed to sit on a chair, positioned at the end of table instead of in the cage. Both subjects were trained to remain on the chair while the experimenter held the right hand in front of them. They were then allowed to approach the reward once the hand was lowered (after object and reward placement).

Deviation from test protocol for first test session of Konrad, Mayday, and Figaro

Three birds (Konrad, Mayday, and Figaro) received a 15 minute 'exploration trial' before the first neophobia trial in session 1. These trials were conducted with different objects then used in the rest of the study and no food was provided. As the duration of sessions became excessively long, we decided to discontinue this procedure and switch to the method described in the main text.

Interventions of experimenter

During testing, the subjects were allowed to move freely within the test compartment. However, on some occasions the experimenter was required to intervene by repositioning the birds at the starting position. These incidences occurred when the birds began to destroy delicate equipment (e.g., safety covers of electrical sockets, video camera), landed on the experimenter, or flew onto the floor.

R^2 AND ICC FORMULAE

$$marginalR^2 = \frac{Var.Fix}{(Var.Fix + Var.Ind + Var.Obj + Var.Res)}$$

$$conditionalR^2 = \frac{(Var.Fix + Var.Ind + Var.Obj)}{(Var.Fix + Var.Ind + Var.Obj + Var.Res)}$$

$$unadjustedICC_{full} = \frac{(Var.Ind + Var.Obj)}{(Var.Fix + Var.Ind + Var.Obj + Var.Res)}$$

$$unadjusted - ICC_{individual} = \frac{Var.Ind}{(Var.Fix + Var.Ind + Var.Obj + Var.Res)}$$

$$unadjusted - ICC_{object} = \frac{Var.Obj}{(Var.Fix + Var.Ind + Var.Obj + Var.Res)}$$

$$single - adjusted - ICC_{individual} = \frac{Var.Ind}{(Var.Ind + Var.Obj + Var.Res)}$$

$$single - adjusted - ICC_{object} = \frac{Var.Obj}{(Var.Ind + Var.Obj + Var.Res)}$$

$$double - adjusted - ICC_{individual} = \frac{Var.Ind}{(Var.Ind + Var.Res)}$$

$$double - adjusted - ICC_{object} = \frac{Var.Obj}{(Var.Obj + Var.Res)}$$

Figure S1: Formulae used for R^2 and ICC values. Variable are defined as follows: Var.Fix = variance of fixed effects; Var.Ind = variance of random effect of individual; Var.Obj = variance of random effect object; Var.Res = residual variance.

MODEL ASSUMPTIONS

Collinearity - Variance Inflation Factors:

Model 1: Individual neophobia score

control	1.052
object.size	1.016
group	1.000
z.object.seq	1.024

Model 2: Trials until habituation

object.size.rl	1.016
group	1.002
z.object.seq	1.024
z.control	1.053
sex	1.002

Model 3: Neophobia and motivation success

z.log_neo_score	1.029
z.session	1.029

Model 4: Neophobia and problem-solving

z.log_neo_score	1.029
z.session	1.096
z.motivation	1.066

Distributions of random effects

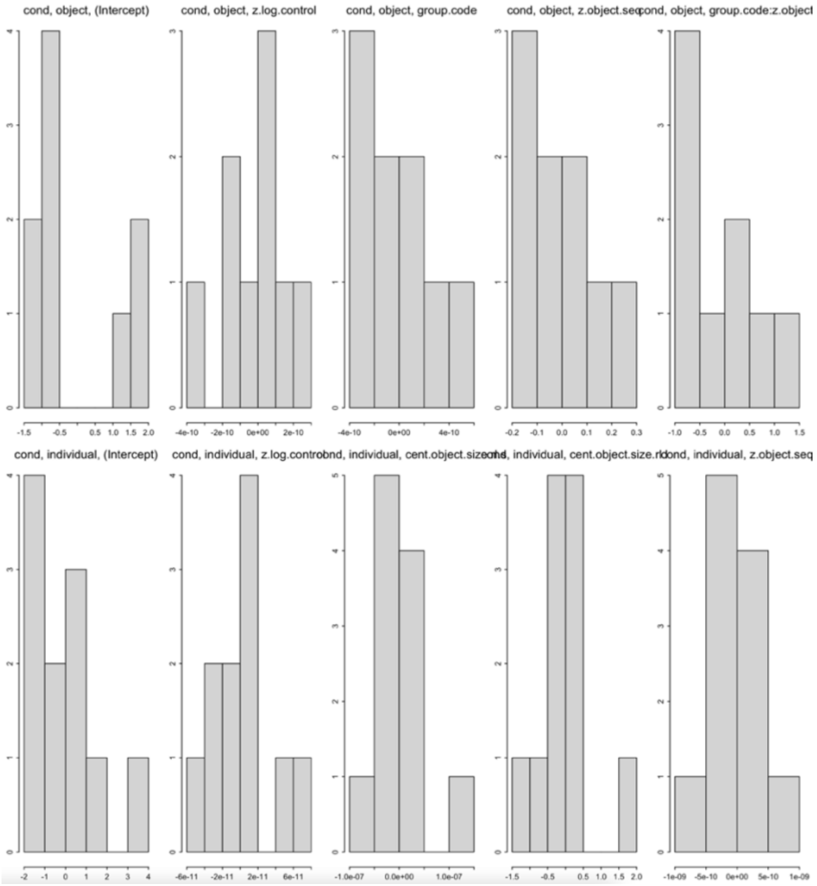

Figure S2: Best Linear Unbiased Predictors (BLUPs) of model 1. Effects on the latency to feed in neophobia trial 1.

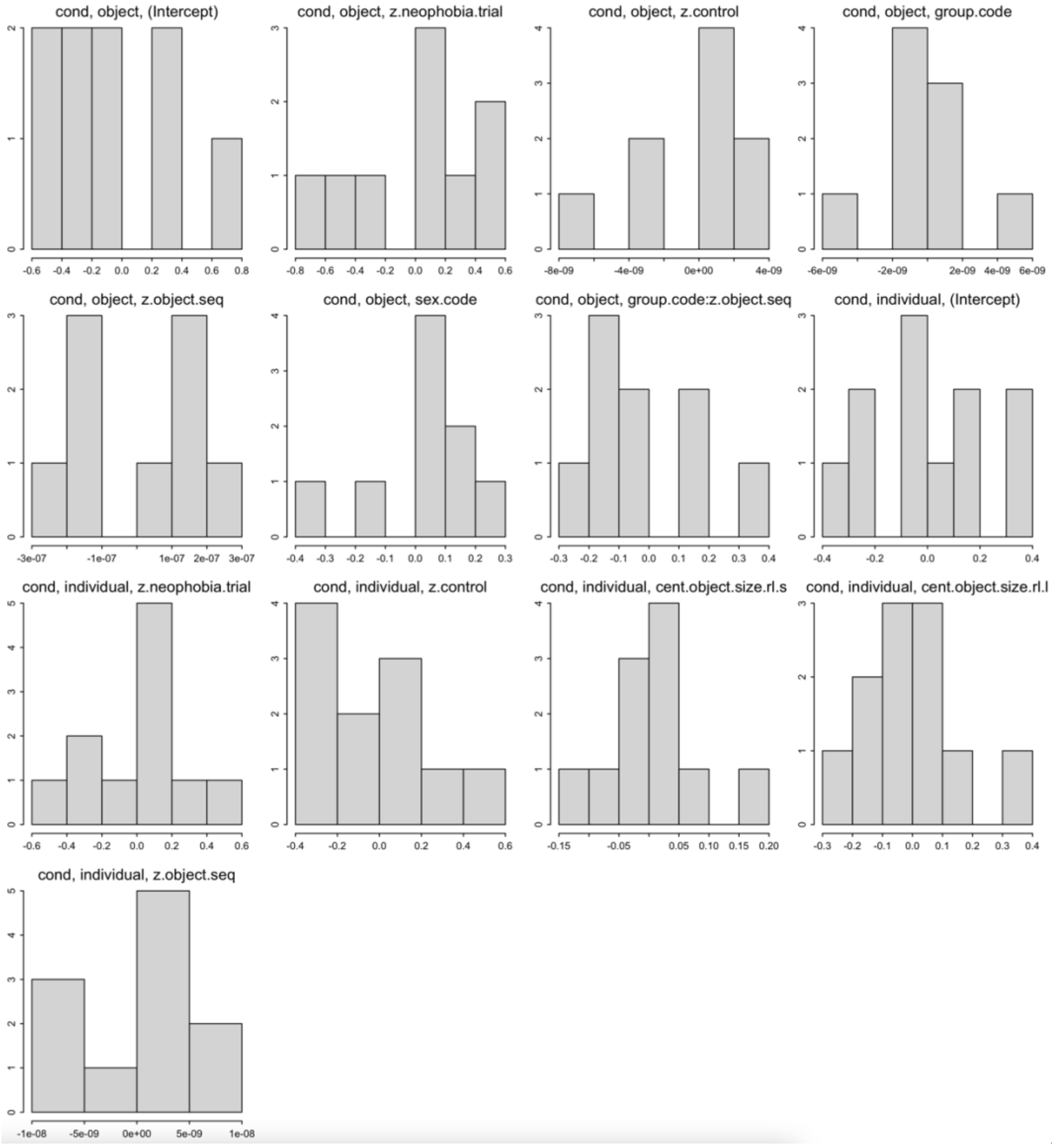

Figure S3: Best Linear Unbiased Predictors (BLUPs) of model 2. Trials until habituation.

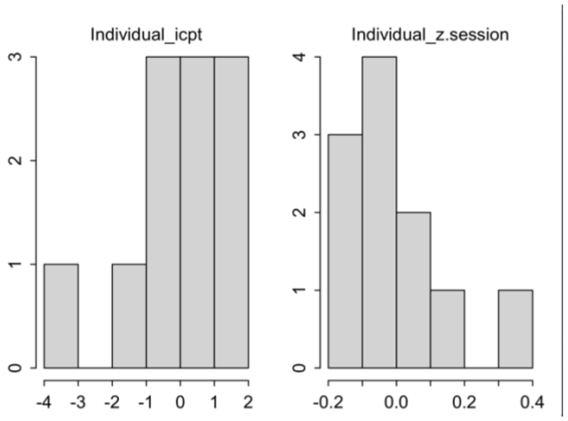

Figure S4: Best Linear Unbiased Predictors (BLUPs) of model 3. Neophobia and motivation.

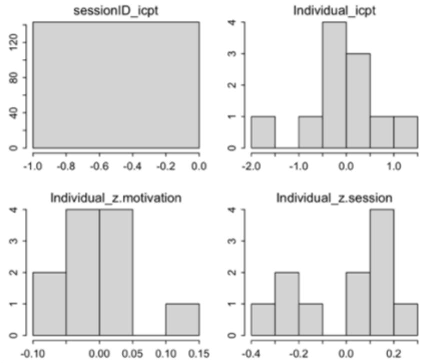

Figure S5: Best Linear Unbiased Predictors (BLUPs) of model 4. Neophobia and problem-solving success

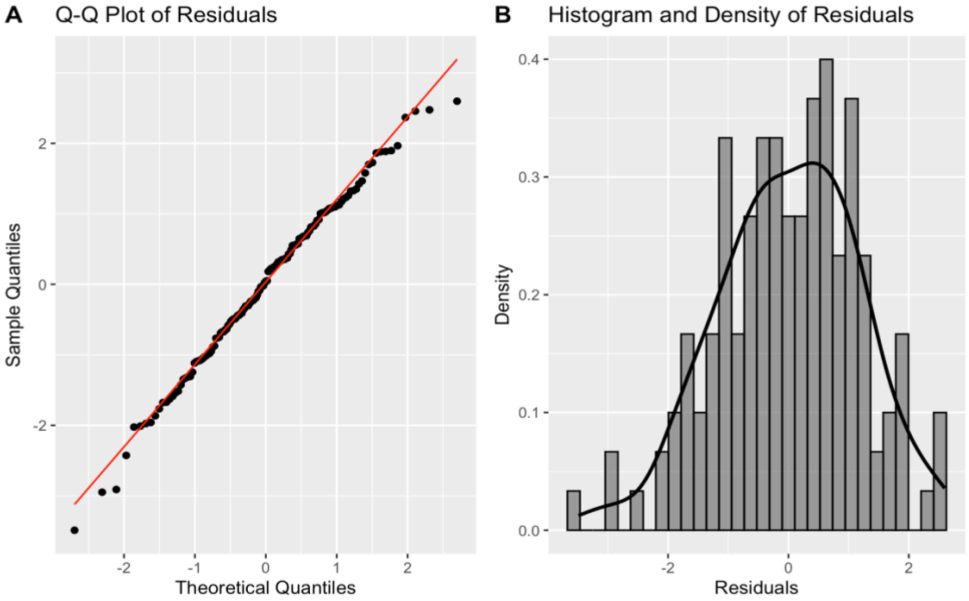

Figure S10: Check for normality of residuals of model 3 (Neophobia and motivation).

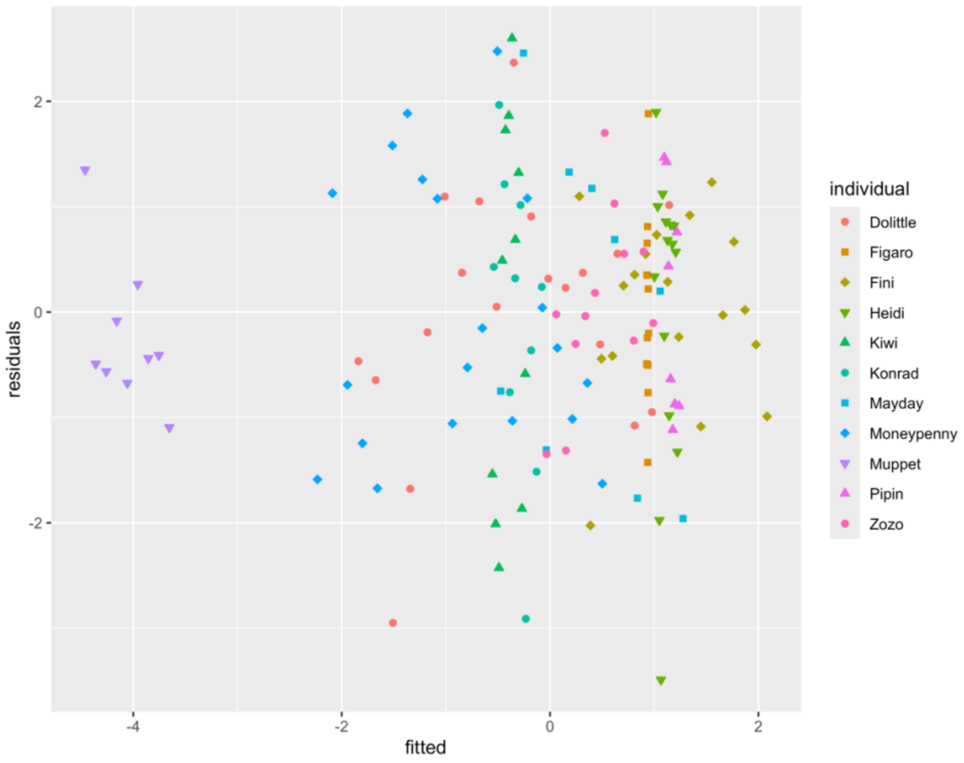

Figure S11: Check for homogeneity of residuals of model 3 (Neophobia and motivation). Shapes and colors depict individuals. Data diverging from ideal distribution can be attributed to one individual Muppet.
